# Evaluation of Cinnamon Essential Oil and Its Emulsion on Biofilm-Associated Components of *Acinetobacter baumannii* Clinical Strains

**DOI:** 10.3390/antibiotics14010106

**Published:** 2025-01-19

**Authors:** Tea Ganić, Ilinka Pećinar, Biljana Nikolić, Dušan Kekić, Nina Tomić, Stefana Cvetković, Stefana Vuletić, Dragana Mitić-Ćulafić

**Affiliations:** 1 Faculty of Biology, University of Belgrade, Studentski trg 16, 11000 Belgrade, Serbia; biljanan@bio.bg.ac.rs (B.N.); stefana.cvetkovic@bio.bg.ac.rs (S.C.); stefana.d@bio.bg.ac.rs (S.V.); mdragana@bio.bg.ac.rs (D.M.-Ć.); 2 Faculty of Agriculture, University of Belgrade, Nemanjina 6, 11080 Belgrade, Serbia; ilinka@agrif.bg.ac.rs; 3 Faculty of Medicine, Institute of Microbiology and Immunology, University of Belgrade, 11000 Belgrade, Serbia; dusan_vk@yahoo.com; 4 Group for Biomedical Engineering and Nanobiotechnology, Institute of Technical Science of SASA, Kneza Mihaila 35/IV, 11000 Belgrade, Serbia; nina.tomic@itn.sanu.ac.rs

**Keywords:** cinnamon essential oil, emulsion, *Acinetobacter baumannii*, biofilm, Raman spectroscopy, gene expression

## Abstract

Background/Objectives: *Acinetobacter baumannii*, one of the most dangerous pathogens, is able to form biofilm structures and aggravate its treatment. For that reason, new antibiofilm agents are in need, and new sources of antibiofilm compounds are being sought from plants and their products. Cinnamon essential oil is associated with a wide spectrum of biological activities, but with a further improvement of its physicochemical properties it could provide even better bioavailability. The aim of this work was the evaluation of the antibiofilm properties of cinnamon essential oil and its emulsion. Methods: In order to evaluate the antibiofilm activity, crystal violet assay was performed to determine biofilm biomass. The main components of the biofilm matrix were measured as well as the motile capacity of the tested strains. Gene expression was monitored with RT-qPCR, while treated biofilms were observed with Raman spectroscopy. Results: A particularly strong potential against pre-formed biofilm with a decreased biomass of up to 66% was found. The effect was monitored not only with regard to the whole biofilm biomass, but also on the individual components of the biofilm matrix such as exopolysaccharides, proteins, and eDNA molecules. Protein share drops in treated biofilms demonstrated the most consistency among strains and rose to 75%. The changes in strain motility and gene expressions were investigated after the treatments were carried out. Raman spectroscopy revealed the influence of the studied compounds on chemical bond types and the components present in the biofilm matrix of the tested strains. Conclusions: The results obtained from this research are promising regarding cinnamon essential oil and its emulsion as potential antibiofilm agents, so further investigation of their activity is encouraged for their potential use in biomedical applications.

## 1. Introduction

*Acinetobacter baumannii* is a biofilm-forming, hospital-acquired infectious pathogen. Its ability to form a biofilm community in different surroundings enables its survival under harsh conditions, while simultaneously, it promotes the transmission of antibiotic resistance among different strains within hospitals. Furthermore, biofilms are responsible for many infections which are difficult or impossible to treat. Cells coated in biofilm matrix can survive for a prolonged period of time under the influence of antimicrobials, which is why this phenomenon is called “biofilm resistance” [1]. Biofilm formation is a complex process involving numerous chemical and biological reactions. It begins with bacterial adherence to biotic or abiotic surfaces, moving from planktonic to sessile life forms [2]. *A. baumannii* exhibits motility, a key virulence factor for biofilm formation [3]. This process is regulated by quorum sensing (QS) via the AbaI/AbaR system, which synthesizes acylhomoserine lactones (AHL) [4]. Bacterial cells within the biofilm are coated with extracellular polymeric matrix produced by bacterial cells. This mainly consists of water (up to 90%) and the rest of the components are proteins, exopolysaccharides (EPS), lipids, and extracellular DNA (eDNA), all found in the *A. baumannii* biofilm matrix. These compounds are very important for biofilm formation and its stability [5]. EPS, known as the biofilm matrix base on which other molecules are attached, is the most dominant constituent of the *A. baumannii* matrix and presents a group of all saccharides outside the cell walls. One of the confirmed saccharides in the *A. baumannii* biofilm is a poly-(1-6)–N-acetylglucosamine (PNAG), conserved in clinical isolates and essential for biofilm formation, adhesion, and protection [6]. Extracellular proteins, including the biofilm-forming protein (Bap), play key roles in the biofilm matrix. Bap, a large surface protein homologous to *S. aureus* Bap, aids in intercellular adhesion, water channel formation, and hospital persistence [7,8]. eDNA, a universal biofilm component, is active within the structure, though its origin and release mechanism remain unclear. As an anionic molecule, it can bind to cationic ones from the environment, and it is involved in horizontal gene transfer, providing a nutrient source during starvation that also enables the penetration of different antimicrobials [5,9].

As mentioned, biofilm formation presents a major problem influencing different aspects of human life, causing serious chronic infection in patients. When a biofilm reaches the maturity stage, it is almost ineradicable by conventional antimicrobial agents. Therefore, it is very important to find novel strategies to combat and control biofilms. In light of this, plants and plant products seem to be useful alternatives, since they have been used for a long time to treat different diseases [10]. Cinnamon is a widely used plant, rich in secondary metabolites [11]. In traditional medicine, it was very popular over the centuries, as was its essential oil. Despite the wide spectrum of the biological activities of cinnamon essential oil, its applicability is limited due to the following inherent characteristics of essential oils: instability in the surrounding environment, susceptibility to high temperature and UV light, and poor deliverability in living tissues due to high polarity [12,13,14]. However, essential oil emulsifications are seen as a way to overcome these problems, at least in increasing the deliverability of active essential oil components to the bacterial cells and enhancing their penetration through the cell wall. Due to the increase in its bioavailability and stability in the form of an emulsion, cinnamon could be beneficial in enhancing antimicrobial and antibiofilm activity [15].

In light of the abovementioned, our previous work [16] chemically characterized cinnamon essential oil (CEO), formulated an emulsion (EM), and provided its physicochemical properties. The determined dominant constituents of the CEO were trans-cinnamaldehyde, cinnamyl acetate, and eugenol. Concerning the EM features, the size distribution range of the droplets were determined to span both nano and micro scales. Also, antibacterial and antibiofilm activities were determined on the selected clinical *A. baumannii* strains, along with their cytotoxic and genotoxic potential [16]. However, numerous questions have been posed by this work and in order to clarify them, further investigation, covered in this work, was conducted. The goal of this study was to deepen our understanding of the already confirmed antibiofilm activity of CEO and EM, focusing on other *A. baumannii* clinical isolates. To do so, we have evaluated their antibacterial and antibiofilm potential, monitored their activity on the most abundant matrix components, namely EPS, proteins, and eDNA, as well as on bacterial motility and the expression of selected genes. The chosen genes were *csu*A and *pil*A, both involved in the motility system’s crucial first step of biofilm formation—adherence. The third gene was *aba*I, which is important for biofilm formation and stability. For the final gene, Raman spectroscopy was performed in order to define the types of chemical bonds and molecules involved in the biofilm matrix and to reveal the effects of CEO and EM activity on them.

## 2. Results

### 2.1. Microdilution Assay

The results obtained from the microdilution assay showed the potentially good antibacterial activity of CEO and EM on all tested isolates. Concerning CEO as a treatment, the minimal inhibitory concentrations (MIC-s) were determined to be 0.5, 0.25, and 1 mg/mL for GN 189, GN 242, and GN 1105, respectively, while the MIC value determined for EM was the same for all tested strains (0.125 mg/mL).

### 2.2. Antibiofilm Assays

**Figure 1:**
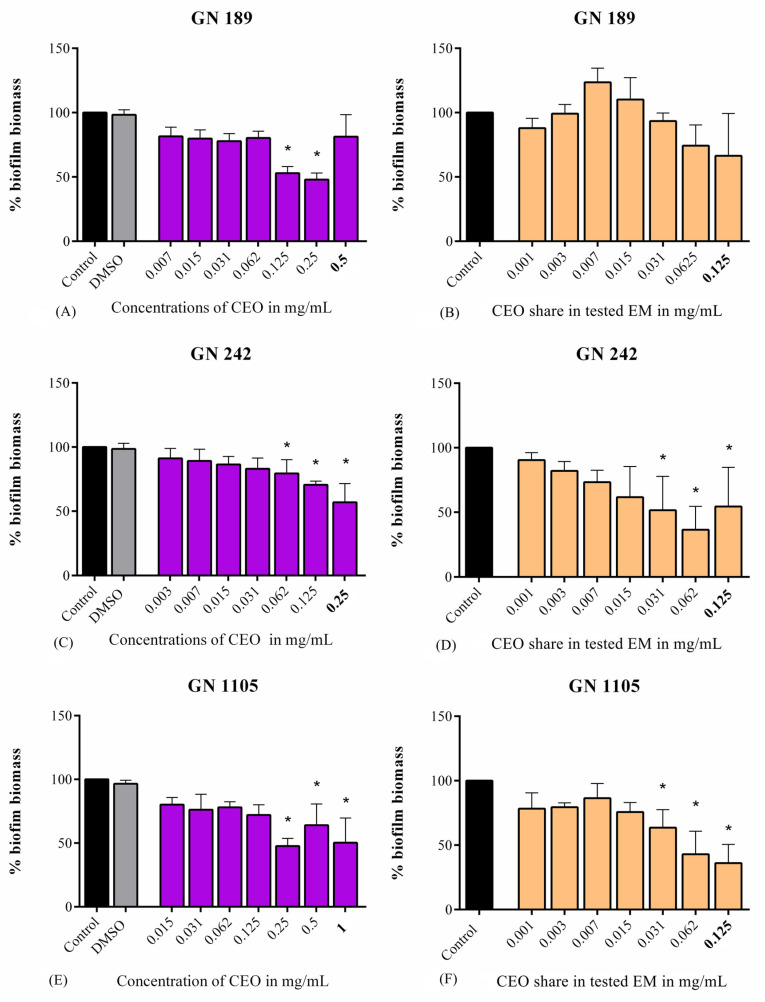
Antibiofilm activity of CEO and EM against *A. baumannii* clinical isolates GN 189 (**A**,**B**), GN 242 (**C**,**D**), and GN 1105 (**E**,**F**). Bolded values represent MIC concentrations. Statistical significance was estimated according to the negative control for EM or DMSO for CEO using one-way ANOVA, Dunnet’s post hoc test. The threshold was estimated to be * *p* < 0.05.

The effects of CEO and EM on biofilm formation of the three clinical isolates were presented as a percentage of the total biofilm biomass (Figure 1). Both CEO and EM showed a trend of decreasing biofilm biomass in all tested strains. The highest reduction in biofilm biomass was observed in GN 1105. EM on MIC and 1/2 MIC concentrations expressed an inhibition of biofilm formation up to 65% and 58%, respectively (Figure 1F). CEO as a treatment was also very effective at the highest tested concentration (MIC), with a recorded statistically significant biofilm inhibition of up to 50% (Figure 1E). In addition, CEO and EM showed biofilm inhibition in a dose-dependent manner in the GN 242 strain. Statistically significant decreases in biomass were detected at the three highest concentrations (1/4 MIC, ½ MIC, and MIC) in biofilms treated with both CEO and EM (Figure 1C,D), and their inhibitions were in the ranges of 9–44% and 10–64% for CEO and EM, respectively. Strain GN 189 expressed lower sensitivity to CEO and EM (Figure 1A,B). The highest biofilm inhibition was observed at subinhibitory concentrations, 1/4 MIC and 1/2 MIC, both experiencing an inhibition of 52% with CEO as a treatment, and up to 34% in the case of EM. Exclusive to this case, the lower tested EM concentrations (0.003–0.015 mg/mL) slightly increased the percentage of biofilm biomass.

**Figure 2:**
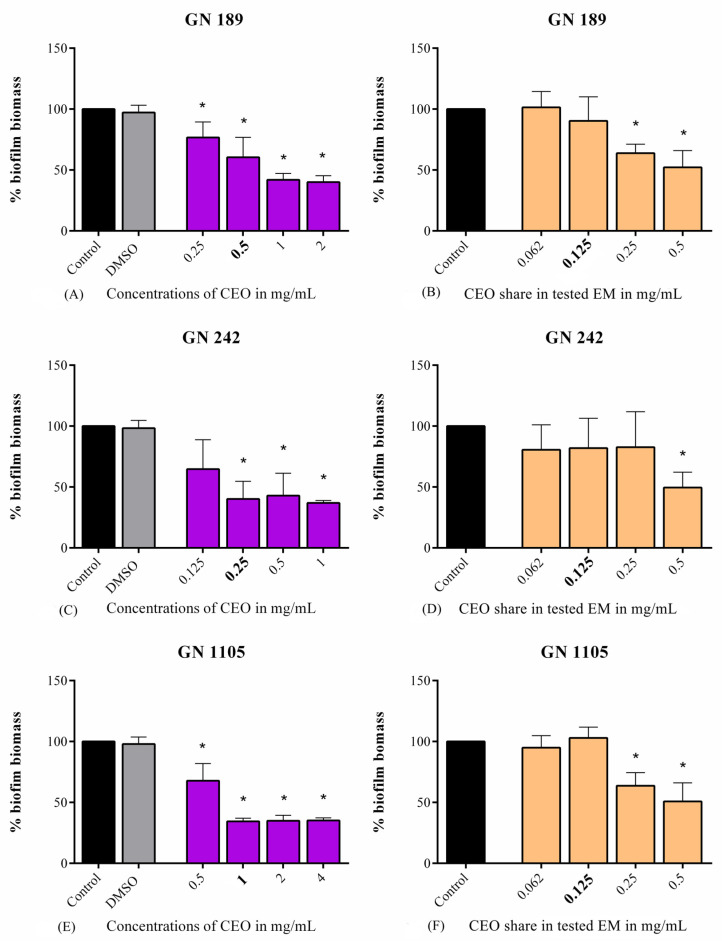
The effect of CEO and EM on the already-formed biofilm of *A. baumannii* clinical isolates GN 189 (**A**,**B**), GN 242 (**C**,**D**), and GN 1105 (**E**,**F**). Bolded values represent MIC concentrations. Statistical significance was estimated according to the negative control for EM or DMSO for CEO using one-way ANOVA, Dunnet’s post hoc test. The threshold was estimated to be * *p* < 0.05.

When it comes to the effect of CEO and EM on the destruction of already-formed biofilms, a decrease in biofilm biomass in a dose-dependent manner was observed in all tested strains. Very noticeable effects were recorded with CEO as the treatment on the GN 189 (Figure 2A) and GN 1105 (Figure 2E) strains, with the ranges of biofilm biomass disruption determined as 24–60% and 33–66%, respectively. Regarding the GN 242 strain (Figure 2C), only the lowest tested concentration was not statistically significant, while biofilm eradication with the highest tested concentration was up to 64% for the treatment with CEO. When biofilm was treated with EM, the highest tested concentrations in all strains were strong enough to reduce biofilm biomass up to 49% for GN 189, 51% for GN 242, and 50% for the GN 1105 strain.

### 2.3. Total Share of EPS

**Figure 3:**
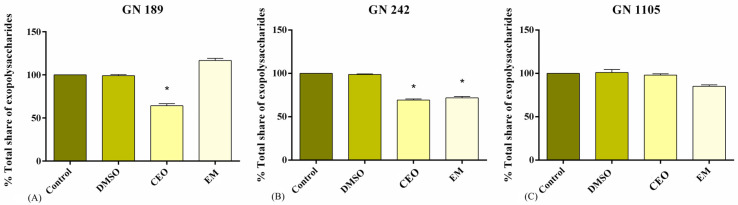
Total share of exopolysaccharides isolated from the biofilm matrix of *A. baumannii* clinical isolates GN 189 (**A**), GN 242 (**B**), and GN 1105 (**C**). The tested concentrations of CEO and EM are the MIC values of GN 189 (0.5 mg/mL for CEO and 0.125 mg/mL for EM), GN 242 (0.25 mg/mL for CEO and 0.125 mg/mL for EM), and GN 1105 (1 mg/mL for CEO and 0.125 mg/mL for EM).The statistical significance was estimated according to the negative control for EM or DMSO for CEO, and by using one-way ANOVA, Dunnet’s post hoc test. The statistically significant threshold was * *p* < 0.05.

The effect of CEO and EM on EPS content within the biofilm biomass was different and strain-specific (Figure 3). CEO treatment led to a reduction in the EPS present in the biofilm matrix of strains GN 189 (up to 37%) and GN 242 (up to 21%), but it did not change their content in the GN 1105 strain. Treatment with EM led to a decrease in EPS only in GN 242 (up to 29%), had no effect on GN 1105, and increased EPS in the case of the GN 189 strain (up to 16%).

### 2.4. Total Share of Proteins

**Figure 4:**
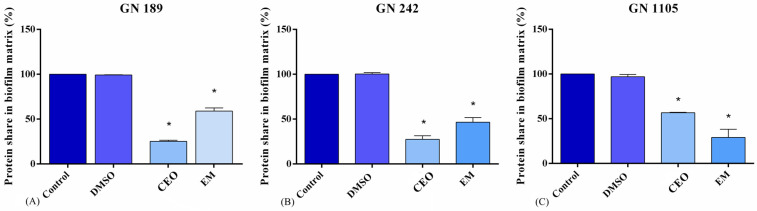
Total share of proteins extracted from biofilm matrix *A. baumannii* clinical isolates GN 189 (**A**), GN 242 (**B**), and GN 1105 (**C**). The tested concentrations of CEO and EM are the MIC values of GN 189 (0.5 mg/mL for CEO and 0.125 mg/mL for EM), GN 242 (0.25 for CEO and 0.125 mg/mL), and GN 1105 (1 mg/mL for CEO and 0.125 mg/mL). Statistical significance was defined by comparing treatments with the negative control for EM or DMSO for CEO, using one-way ANOVA, Dunnet’s post hoc test. The threshold was estimated to be * *p* < 0.05.

A glance at the overall results in this experiment showed a similar trend in all tested strains (Figure 4). The total share of proteins decreased after treatment compared to the control. Therefore, a generally strong effect of both CEO and EM on proteins was noticeable. The same trends were recorded on strains GN 189 and GN 242, where the MIC values of CEO had a slightly stronger effect than EM. The reduction in the total share of proteins treated with CEO was up to 75% and 73% for GN 189 and GN 242, respectively, and 42% and 54% when treated with EM. A slightly different response to the treatments was shown in the case of strain GN 1105, where a stronger decrease was obtained with EM treatment (71%) than CEO treatment (44%).

### 2.5. Concentrations of eDNA

The concentrations of isolated eDNA from the biofilm matrix are presented in Table 1. For strain GN 189, both CEO and EM increased eDNA concentrations. In the case of EM, concentration was higher by more than two-fold compared to the control. Furthermore, for the GN 242 strain, CEO treatment slightly decreased eDNA, but EM almost doubled it. For strain GN 1105, EM had almost no influence, while CEO increased the eDNA concentration.

### 2.6. Motility Assay

**Figure 5:**
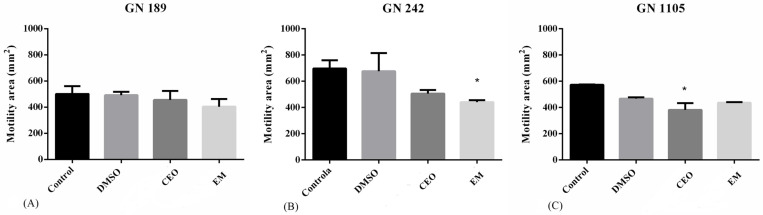
The effects of CEO and EM on the motility of strains GN 189 (**A**), GN 242 (**B**), and GN 1105 (**C**). The tested concentrations of CEO and EM are the MIC of GN 189 (0.25 mg/mL for CEO and 0.062 mg/mL for EM), GN 242 (0.125 for CEO and 0.062 mg/mL), and GN 1105 (0.5 mg/mL for CEO and 0.062 mg/mL). Statistical significance was determined according to the negative control for EM or DMSO for CEO, performed using one-way Anova, Dunnet’s post hoc test, while the threshold was * *p* < 0.05.

The motile capacity of tested strains after the treatment with CEO and EM is presented in Figure 5. In all tested strains, the same decreasing motility trend in treated samples is visible, but not always with statistical significance. The least sensitive and the least motile strain was GN 189, where no significant decrease was detected. Furthermore, much stronger effects were noted in the GN 242 strain, where EM expressed a statistically significant decrease in motility. Also, it is worth mentioning that GN 242 is the most motile strain among all three strains according to motility area. Concerning the GN 1105 strain, CEO exhibited a stronger and statistically significant activity compared to EM.

### 2.7. RT-qPCR

**Figure 6:**
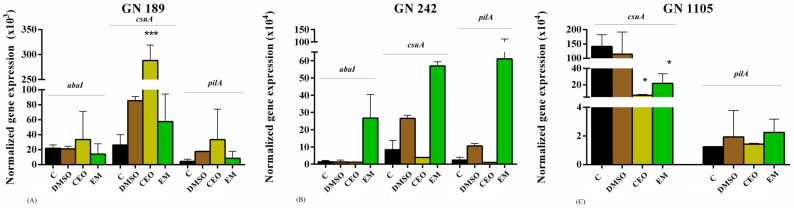
Gene expression analysis of the *A. baumannii* biofilm of clinical isolates GN 189 (**A**), GN 242 (**B**), and GN 1105 (**C**), treated with CEO and EM. The tested concentrations of CEO and EM are the MIC values of GN 189 (0.5 mg/mL for CEO and 0.125 mg/mL for EM), GN 242 (0.25 for CEO and 0.125 mg/mL), and GN 1105 (1 mg/mL for CEO and 0.125 mg/mL). The results are presented as the normalized gene expression. Statistical significance regarding the negative control (C in graphs) for EM and DMSO for CEO was determined using one-way ANOVA with Dunnet’s post hoc test and the threshold was * *p* < 0.05, *** *p* < 0.001.

In order to deepen our understanding and propose the possible molecular pathways of CEO and EM activity on the already-formed biofilms, the expression of genes *aba*I, *csu*A, and *pil*A was monitored (Figure 6). All of the genes were detected in the tested isolates, with the exception of the *aba*I gene which was not present in the GN 1105 strain. Concerning the GN 189 strain, an evident increase in gene expression was observed after biofilm treatment with CEO, but a statistically significant change was present only in the case of the *csu*A gene. In the case of EM treatment, an opposite but nonsignificant effect was observed. When it comes to strain GN 242, up-regulated gene expressions were detected after EM treatment, while CEO induced a reducing but nonsignificant effect. Finally, for strain GN 1105, *csu*A gene expression was significantly diminished after both treatments, while the effect on *pilA* was negligible.

### 2.8. Raman Spectroscopy

**Figure 7:**
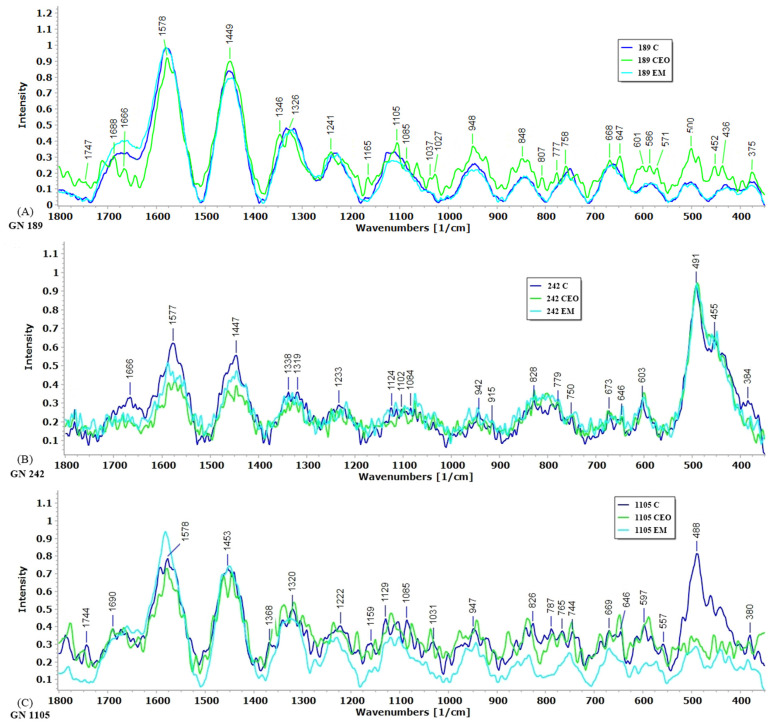
Averages of normalized Raman spectra of the *A. baumannii* biofilm matrix: GN 189 (**A**), GN 242 (**B**), GN 1105 (**C**); (C—control, CEO–cinnamon essential oil, EM—cinnamon emulsion). Spectral range is from 350 to 1800 cm^−1^.

Raman spectroscopy was performed on mature biofilm, treated with CEO and EM, as well as on the untreated control. Every Raman band corresponds and may suggest the type of chemical structure or molecule (Figure 7). The most intense peaks of the Raman spectrum can be observed in the biofilm of all three tested strains. These peaks were detected at 1577–1578 cm^−1^, 1447–1453 cm^−1^, and around 488–550 cm^−1^. The intensities of the peaks around 1578 cm^−1^ in the strains GN 242 and GN 1105 were different. Regarding the GN 242 strain, the highest peak intensity was observed in the control biofilm, while for the GN 1105 strain, the highest peak was observed in the biofilm treated with EM. A similar response was observed for the peaks around 1447–1453 cm^−1^. In the GN 189 strain, the region around 488–550 cm^−1^ had peaks with low intensities compared to the other two strains. Furthermore, in the GN 1105 strain, the difference between the peak intensity of the control and treatments was much more pronounced.

**Figure 8:**
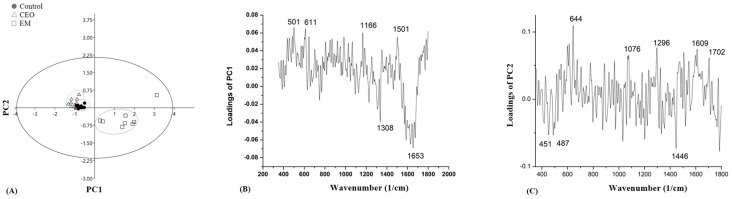
PC analysis of score plots (**A**) and loading plots (**B**,**C**) obtained from the Raman spectra of the *A.baumannii* GN 189 biofilm.

In order to find a difference between the biofilms of the control, CEO, and EM treatments, Principal Component Analysis (PCA) was applied on every tested strain. The score plots and loading plots gathered from the PCA for strain GN 189 are presented in Figure 8. A clear separation between the biofilm treated with EM, the control, and the CEO-treated biofilm is visible (Figure 8A). The biofilm treated with EM is separated according to both the PC1 (40.1%) and PC2 (12.05%) axes. The most intense peak obtained from Figure 8B, which separates the emulsion-treated biofilm from the rest, was observed at 1653 cm^−1^. According to Figure 8C, the most intense peaks were at 644 cm^−1^ and 1446 cm^−1^.

**Figure 9:**
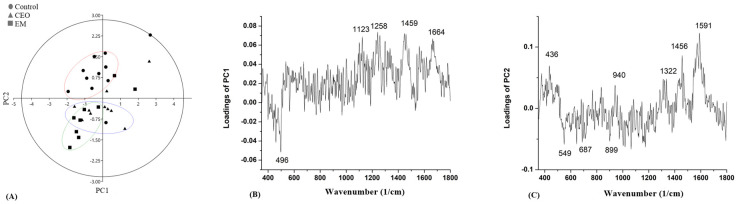
PC analysis score plots (**A**) and loading plots (**B**,**C**) obtained from the Raman spectra of the *A.baumannii* GN 242 biofilm.

Separation in strain GN 242 was low based on the results obtained from the PC analysis (Figure 9A). The percentage of disjunction toward the PC1 axis was 28.45%, and, according to PC2, 11%. The most intense peak based on the PC1 loading, which mainly separates biofilm treated with CEO from the control and EM-treated ones, was determined at 496 cm^−1^, while the most intense peak according to Figure 9C was at 1591 cm^−1^.

**Figure 10:**
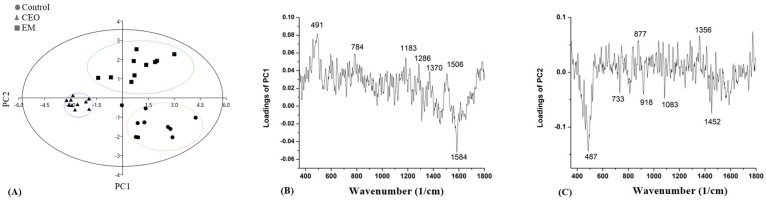
PC analysis score plots (**A**) and loading plots (**B**,**C**) obtained from the Raman spectra of the *A.baumannii* GN 1105 biofilm.

The score plots and loading plots for strain GN 1105 are presented in Figure 10. According to Figure 10A, separations of PC1 and PC2 were moderate. Separation based on the PC1 axis was approximately 23.82% and this distinguished the biofilm treated with CEO from the control one and the biofilm treated with EM. Furthermore, the separation on PC2 between the control, the biofilm treated with CEO, and the biofilm treated with EM was 9%. The most intense peak obtained from Figure 10B was at 1584 cm^−1^, while in Figure 10C this was at 487 cm^−1^.

## 3. Discussion

Many infections are caused by different pathogenic bacteria able to form a biofilm on different tissues and/or medical implants. When a biofilm is formed, conventional treatments are usually ineffective or a prolonged period of curing is necessary, leading to serious burden to healthcare systems and huge economic losses. Furthermore, biofilm-associated infections notably contribute to the increase in health disorders and mortality rate. For these reasons, it is a top priority to find a way to efficiently eradicate and treat biofilm infections [17]. *Acinetobacter baumannii* is a pathogenic species causing serious concerns over its ability to adapt in a wide range of different surroundings. One of its survival strategies is biofilm formation, which enables the evasion of antibacterial drugs and its persistence under the unfavorable environmental conditions [18]. The urgent need for a new antibiofilm agent is very important due to the insufficient efficacy of conventional antibiotics against a growing number of resistant pathogens. With that in mind, research for alternative therapeutics is of utmost importance. In light of this, plant products have been proven to be effective antibiofilm agents where their potential ability to inhibit formation or disrupt already-formed biofilm is concerned [19]. Unfortunately, the lack of knowledge on this topic is evident, so a better understanding of the underlying mechanisms of these types of antibiofilm agents is required. Cinnamon essential oil is a natural, volatile compound with proven antibacterial and antibiofilm potential [20]. However, the application of the different essential oils, including CEO, is limited due to its physicochemical properties, which is why the synthesis of EM may improve its bioactivity. Natural compounds, such as oily ones, could have a better cell wall permeability in the form of an emulsion. Moreover, emulsification enables controlled release of active molecules [21].

In this study, we have investigated the biofilm of the *A. baumannii* clinical isolates and the antibiofilm potential of CEO and EM against it. As part of the pre-screening test, the antibacterial activity of CEO and EM was evaluated. A moderate inhibitory potential was shown for CEO, while for EM, the MIC values were much lower and uniform between strains. In agreement with our results [22], similar results from testing cinnamon as an essential oil against the *A. baumannii* ATCC 19606 strain were obtained. Moreover, one of the most dominant components of cinnamon essential oil, cinnamaldehyde, expressed an antibacterial activity against *A. baumannii* clinical strains in the same MIC ranges as our results [23]. The wide spectrum of different bacterial species has been proven to be sensitive against cinnamon essential oil with different MIC values [24]. Variations in the biological activity of cinnamon essential oil and emulsions exist between studies, but this can be explained due to differences among the tested cinnamon species, the parts of the plants that have been used as their source, geographical distribution, and seasonal features, etc. Furthermore, different types of methods were used to extract the essential oil and synthesize the emulsion, which can directly influence their antibacterial properties [25].

Furthermore, natural compounds, such as essential oils, can act as antibiofilm agents and influence different phases of biofilm formation. The most sensitive stage is the first step, which involves the initial adhesion of the bacterial cells on the appropriate surface [26]. For that reason, the first part of our antibiofilm research focused on the inhibition of the biofilm formation of the tested clinical strains. Both CEO and EM exhibited very good activity in terms of inhibiting biofilm formation and reducing the overall biomass within it. Statistically significant biofilm formation inhibition was observed in subinhibitory concentrations as well as on the MIC values of most of the strains. Moreover, EM was effective as a treatment, even in a lower concentration range than CEO. The antibiofilm activity of cinnamon essential oil and extracts tested on a different bacterial species [27,28] was very noticeable and even stronger compared to our results. According to the results of Kim et al. [29], cinnamon bark essential oil exhibited antibiofilm activity, while isolated cinnamaldehyde, as the most abundant component, exhibited the strongest antibiofilm activity among all isolated oil components. In light of this and based on our previous work [16], the chemical analysis of our CEO showed that cinnamaldehyde was also the most dominant component. Therefore, the antibiofilm efficacy of CEO and EM can be attributed to the strong activity of the most dominant component. Both cinnamaldehyde and its nano-emulsion expressed a notable, or even stronger, antibiofilm potential [30]. Moreover, the obtained results showing a more efficient antibiofilm activity on subinhibitory concentrations could be explained due to the interactions between active components within treatments. These interactions could lead to QS inhibition, a decrease in EPS production, or changes in the bacterial cell metabolism [31,32]. In the continuation of the experiment, the activity of the tested compounds on already-formed biofilms was monitored. Despite the fact that such experiments have rarely been conducted, Topa et al. [33] have demonstrated the very strong activity of cinnamaldehyde on already-formed biofilm of *Pseudomonas aeruginosa*. Isolated natural compounds have demonstrated successful biofilm disruption on the *S. aureus* and *Staphylococcus epidermidis* species [34]. Furthermore, myrtenol, expressed very good potential in disrupting the already-formed biofilm of *A. baumannii* strains in subinhibitory concentrations [35]. All of these promising results could be explained, at least partially, by the fact that the essential oil components can interfere with electron transport and protein translocation on the cell membrane, but they could also influence cell components and their synthesis, thus leading to cell death. Also, cinnamaldehyde can diffuse through the biofilm matrix and disrupt its consistency [36].

Sessile forms of bacterial cells are coated with a biofilm matrix that consists of EPS, proteins, eDNA, and lipids. The prime role of the matrix is to serve as a barrier protecting cells from antimicrobial agents and other adverse environmental conditions. The most prevalent extracellular polymeric substances are usually polysaccharides. Their roles are important, not only as a barrier, but also for the initial attachment and aggregation of bacterial cells and the mechanical stability of the formed biofilm [37]. In this context, we have monitored the change in the EPS constituent of the biofilm matrix after the treatment with CEO and EM. The EPS percentage reduction was visible in almost all strains and treatments. In agreement with our results, Subhaswaraj et al. [38] have demonstrated a decrease in EPS in the biofilm matrix after cinnamaldehyde treatment. Moreover, Selvaraj et al. [35] showed the reduction in polysaccharide content in the *A. baumannii* strains matrix treated with a subinhibitory concentration of myrtenol. Lowering the polysaccharide share in the biofilm matrix is in agreement with the results obtained from antibiofilm assays. With a reduction in EPS, biofilm stability could be disrupted, leading to the more pronounced exposure of bacterial cells to antibiofilm agents. It is also reported that glycoside hydrolases, especially if they are combined with other antimicrobial agents, could degrade polysaccharides into smaller molecules and effectively disturb the matrix [28,39]. One of the identified polysaccharides produced by *A. baumannii* and extracted into the biofilm matrix is PNAG, which helps other bacteria attach to surface or other cells. The disruption of this macromolecule renders the biofilm incapable of maturing.

Furthermore, we have monitored the effect of CEO and EM on protein share within the biofilm matrix. Proteins are a very important part of matrix, responsible for its formation and stability, as well as for the dispersal processes [40]. In our study, CEO and EM decreased the level of proteins in the biofilm matrix in a manner which could be correlated with the results of the antibiofilm experiments. One could speculate that a protein share reduction could implicate the potential degradation of the Bap protein, which is important for both biofilm formation and stability [41]. Furthermore, it was found that the plant extract of *Actinidia deliciosa* decreased the protein concentration in the matrix [42]. The third widespread biofilm matrix component that we have monitored was eDNA. The source of eDNA in the matrix is still under question, but one of the explaining hypothesis points at a cell lysis. Nevertheless, their role in the matrix is very important, as they are involved in horizontal gene transfer, the neutralization of antimicrobial peptides, the restriction of antimicrobial diffusion, and the construction of biofilm spreading [9]. The results of our work suggest that, in most of the cases, CEO and EM led to an increase in the concentration of eDNA molecules. Contrary to our results, it was shown that cinnamon and clove water extract decreased eDNA content in *Listeria monocytogenes* biofilm [43]. Regarding our results, eDNA concentration decreased only in the case of the GN 242 strain treated with CEO, a possible explanation being the antibiofilm’s ability to reduce DNA content. This reduction probably contributed to the disturbed stability of the biofilm and the higher susceptibility of the involved bacterial cells to the antimicrobial agents. Concerning other research results, cardamom essential oil did not change eDNA concentration in the *S. aureus* biofilm matrix [44]; meanwhile, for results more similar to ours [45], eDNA concentrations increased with cephem treatment. The eDNA increase can be potentially explained by the increased cell lysis and enlarged eDNA releases [46].

Furthermore, based on numerous data, the biofilm formation of *Acinetobacter baumannii* is directly correlated with surface-associated motility. With that in mind, we have assumed that treatment with CEO and EM could influence both biofilm formation and the movement of the *A. baumannii* tested strains. Our results suggest that both CEO and EM inhibited strain motilities in similar ways. Compared to results from other studies, virstatin inhibited *A.baumannii* pili production and its migration [47]. Also, curcumin inhibited motility movement in *A.baumannii* strains [48]. It is known that many environmental substances can also influence surface-associated motility, including antibiotics such as kanamycin, ampicillin, or tetracycline, causing a decrease in the motile capability of the strains [49]. A correlation between the inhibition of biofilm formation and the decrease in motility with CEO and EM only proved the connection between the two virulence factors.

For a deeper understanding of the possible mechanisms of antibiofilm activity, the changes in relative genes expression of *aba*I, *csu*A, and *pil*A were monitored after CEO and EM were used as treatments. The results obtained from RT-qPCR showed that the *aba*I gene was not present in all tested strains. It was shown that the presence of the *aba*I gene is not mandatory in every isolate, confirming its attendance in approximately 80% of the tested isolates [4]. On the other hand, the level of *aba*I gene expression in the GN 189 and GN 242 strains confirmed the absence of a statistically significant change. On the other hand, an increased expression was determined in our study, whereby antibiotics increased AdeFGH efflux pump expression, leading to the coefflux of AHL molecules and antibiotics [50]. AHL molecules from the extracellular space increase penetration into bacterial cells, attaching to the AbaR and activating the synthesis of new AHL molecules. Furthermore, *csu*A gene expression was monitored in our work. This gene is a part of a csu operon, encoding Csu pilus, important for adherence on abiotic surfaces [51]. Gene *csu*A was detected in all strains, while its expression was variable and strain- and treatment-dependent. Increased gene expression was observed in the GN 189 strain and GN 242 strain after EM treatment, but a decreased expression was visible in the GN 1105 strain and GN 242 after CEO treatment. This reduction is in line with some other obtained results, such as the ones on antibiofilm and motility. Down-regulated *csuA* gene expression was observed in a few studies, for instance with treatments using human serum albumin or kojic acid [52,53]. Interestingly, previously published data [52] has highlighted that the decreased *csu*A gene expression is followed by a similar same trend in *bfm*R and *bfm*S gene expression. These genes are part of two component systems that control the csu operon. Furthermore, it was noted that combination of subinhibitory concentrations of sulfamethoxazole and trimethoprim inhibited CsuA/B gene expression as well as biofilm formation [54]. Regarding these results, Ref. [32] has shown that geraniol and eugenol can bind to the csuE subunit, which is followed by the inhibition of cell adhesion and biofilm formation. Contrary to this result [55], *csu*A gene expression increased under the influence of the *S.aureus* toxin. Furthermore, the *pil*A gene encodes the PilA subunit, which is part of a type IV pili system. Its function is connected not only to a twitching type of motility, but also to surface adherence and biofilm formation. Generally, genes that encode the type IV pili system are conserved, except for the *pil*A gene [56,57]. Our results only showed increased *pil*A expression in the biofilm of the GN 242 strain treated with EM. In other strains and treatments, gene expression was not significantly changed. Under the influence of polymyxin treatment, *pil*A gene expression was modified in both directions, being up- and down-regulated depending on strain specificity [58]. Interestingly, sub-MIC imipenem concentrations led to the up-regulation of genes important for PilA subunit assembly [59]. In agreement with our results [60], it was previously shown that there is an increase in pilA gene expression under the influence of human pleural fluid. Bearing in mind that bacterial motility is the most pronounced during the initial biofilm-forming step, the increased expression of motility genes in later stages of biofilm formation may destabilize and disturb biofilm maturity [61].

In order to analyze biofilm in depth, Raman spectroscopy was applied as a nondestructive method useful in identifying the chemical compounds and bonds involved in different structures. Even though Raman is widely used, its application in biofilm and antibiofilm studies is still very scarce. All of the identified peaks gathered from the Raman spectroscopy are listed in Table 2. The bands observed in the region from 1577 cm^−1^ to 1591 cm^−1^ point to adenine and guanine ring vibration, while the peaks at 1447–1453 cm^−1^ are attributed to CH_2_ deformation, which could be found in lipids and proteins [62]. The region in Raman spectra from 380 to 550 cm^−1^, in all tested isolates, are bands assigned to the polysaccharides [63]. The broad region around peaks at 1666 cm^−1^ and 1690 cm^−1^, as well as 1222 cm^−1^ and 1233 cm^−1^, all suggest the presence of proteins. Amide I and C=C stretching correlate with peaks of 1666 cm^−1^ and 1690 cm^−1^ [64], respectively, while 1222 cm^−1^–1241 cm^−1^ are assigned to N–H bending and C–O stretching, belonging to the amide III group of proteins [62,65]. The rest of the detected peaks and their assigned structure are presented in Table 2.

Regarding strain GN 189, a peak assigned at 1449 cm^−1^ suggests a potential presence of lipids or proteins, while the differences in spectral intensities may indicate different responses to CEO and EM treatments. A higher intensity is observed after CEO treatment compared to the control, while lower intensity was observed after EM treatment. Interestingly, only for strain GN 189 was there a visible differentiation of peak intensities around 1666 cm^−1^. This peak, assigned to the protein molecules, could also explain why CEO as a treatment decreased the total share of protein structures. The result is in agreement with spectrophotometric assays, where a statistical significance was observed after CEO treatment. Furthermore, the PC analysis of the GN 189 strain revealed the reasons for separations between the recorded biofilms. The most intense peak detected according to the PC1 analysis is at 1653 cm^−1^, positioned below the axis, implying negative intensity loading. The mentioned peak could be assigned to the protein structure, specifically, the amide I group of amino acids, according to the literature [65]. The most predominant negative intensity loading of PC2, which separates the EM-treated from the two other biofilms, is observed at 1446 cm^−1^, connected to potential CH_2_ deformations. Those chemical bonds could be found in protein or lipid structures [62]. Positive intensity loading above the PC2 axis around 644 cm^−1^ suggests the tyrosine [66] corresponding to the protein structure. Out of all the PC analyses, the results for the GN 189 strain biofilm treated with EM could be singled out due to their specific lipid-protein content. The separation on the PC1 axis implies that a possible differentiation exists, indicating the presence of some specific amino acid (e.g., phenylalanine). According to the results from the Raman spectra for each isolate, it was observed that the lower intensity of peaks (at 1447 cm^−1^) after the CEO and EM treatments of strain GN 242 could correspond to the potential presence of proteins. These results imply protein denaturation or conformational changes could occur, leading to cell death [67]. Moreover, for the same strain, the magnitude of the Raman spectra decreased after CEO treatment, with a peak that corresponds to the DNA structures (1577 cm^−1^), suggesting ring structure deformation [67]. This result is in accordance with the results obtained in eDNA quantification.

**Table 2:** Vibrational bands and their assignments in the average and normalized spectra and the wavelengths obtained from the PC analyses using Raman spectroscopy in the biofilm matrix of *A. baumannii* isolates (GN 189, GN 242, and GN 1105).

Wave Number (cm^−1^)	Vibrational Mode	Chemical Moiety	References
375–603	CC bending in benzene ring; C–C–O bending; C–OH twisting	Carbohydrates	[63,65,68]
549	S-S stretch	Protein	[63]
611	Phenylalanine	Protein	[65]
644–647	C–S stretching and C–C twisting of proteins	Tyrosine, protein	[66]
668–683	Gunanine	DNA/RNA	[62]
733	Adenine	DNA/RNA	[62,63]
744–828	Timine, cytosine, uracil	DNA/RNA	[63]
848	Tyrosine	Protein	[65]
877	C–CH	Protein	[65]
899	Tryptophan	Protein	[66]
915–918	Side group (COH), (C–CH) stretching, (O–CH)	Carbohydrates	[64]
940–948	α 1,3 glucan; Deoxyribose	Carbohydrates	[62,65,69]
1027–1037	Phenylalanine/proline (C–H in plane deformation); CO and CC stretching	Protein	[63]
1063	C–C; C–N bands	Lipid, protein	[65]
1076	PO^2-^ symmetrical stretching	DNA/RNA	[63]
1084; 1085	C–C stretching	Lipids	[64]
1102–1105	PO^2−^ symmetrical stretching	DNA/RNA	[70]
1123–1129	Glucose; C–C, C–N stretching	Carbohydrates; protein	[64,66]
1159–1166 (1183)	C–C stretching; CH_2_ deformation	Lipids	[64]
1222; 1233	N–H bending and CO stretching (amide III) and CN amide (stretching)	Protein	[62]
1241	Amide III	Protein	[65]
1258	CH_2_ deformation	Protein, lipids	[66]
1286	C–N, N–H, Amide III	Protein	[63]
1296–1306	CH_2_ deformation	Protein, lipids	[64]
1319–1326	Tyrosine, CH_2_ deformation	Lipid, protein	[66]
1338	Adenine ring mode and CH_2_ deformation modes (non-aromatic residues), α-helices	DNA/RNA protein	[66]
1346–1370	C–OH stretching	Carbohydrates	[64]
1446–1459	CH_2_ deformation	Lipids, protein	[62]
1501–1506	CH_2_ deformation	Lipids, protein	[64]
1577–1591	A,G	DNA/RNA	[62,63]
1609		Protein	[62]
1653	Amide I	Protein	[65]
1664–1666	C=C, C=O, C–N stretching, N–H binding	Protein	[64,66]
1688–1702	Amide I	Protein	[64]
1744; 1747	C=C, C=O stretching	Carbohydrates, lipids	[68]

The PC analysis of the GN 242 strain reveals an unclear separation between control and treatments. According to the loading plots obtained from PC1, the most intense peak was observed as the negative loading at 496 cm^−1^, and this corresponds to the carbohydrates according to the literature [63,65,68]. A peak at 1591 cm^−1^ showed the highest intensity assigned to potential DNA molecules [62,63] and could imply differences between control and treated biofilms exist, which means that treatments led to the changes in the DNA content of the biofilm matrix.

In a wide range around 300–500 cm^−1^ in strain GN 1105, lower intensities of peaks after the CEO and EM treatments are visible. These peaks correspond to carbohydrates [63,65,68]. A decrease in these peaks after the CEO and EM treatments are in agreement with the results obtained in the spectrophotometric assay measuring EPS share, suggesting that both treatments lowered the amount of carbohydrates in the biofilm matrix. After the PC analysis, the most intense peak obtained from the PC1 loading plot, separating CEO-treated from control and EM-treated biofilms, was at 1584 cm^−1^. This peak is assigned to DNK molecules [62,63]. This result is in agreement with the results of eDNA quantification. The most intense peak gathered from the PC2 loading plot was observed at 487 cm^−1^, suggesting the presence of carbohydrates [63,65,68], which separate the biofilm treated with EM from the other two biofilms.

## 4. Materials and Methods

### 4.1. Chemicals, Reagents, and Media

The CEO was dissolved in 50% dimetilsulfoxide (DMSO) in a concentration of 100 mg/mL and was used as the primary stock solution. CEO was purchased from Frey+Lau GmbH (Henstedt-Ulzburg, Germany). The prepared EM [16] was in a primary concentration of 174 mg/mL. DMSO, Na_2_HPO_4_ × 12H_2_O, and NaH_2_PO_4_ × 2H_2_O were acquired from Centrochem (Stara Pazova, Serbia). MuellerHinton broth (MHB), tryptone soya broth (TSB), and Luria–Bertani broth (LB) were from Titan Biotech Ltd. (Rajasthan, India). Resazurin was from SERVA Electrophoresis GmbH, (Heidelberg, Germany). Cristal violet was from Lach-Ner s.r.o. (Neraotvice, Czech Republic). Isopropanol was from Carlo Erba Reagents (Emmendingen, Germany), while chloroform was from Fisher Chemicals, Thermo Fisher Scientific (Waltham, MA, USA). The TRIzol reagent was from Ambion by Life Technologies, Thermo Fisher Scientific (Waltham, MA, USA). Sodium chloride (NaCl) was from Merck KGaA (Darmstadt, Germany). Phenol, sulfuric acid, sodium acetate, glucose, trichloroacetic acid solution, and Bradford reagent were from Sigma-Aldrich (Steinheim am Albuch, Germany). DNaseRNase free water and proteinase K were from Gibco by Life Technologies (Thermo Fisher Scientific, Waltham, MA, USA). Ethanol and acetone were from Zorka Pharma (Šabac, Serbia).

### 4.2. Bacterial Strains

*Acinetobacter baumannii* clinical strains (GN 189, GN 242, and GN 1105), isolated from the tip of an aspirational catheter, tracheal aspirate, and wound, respectively, were kept in LB media with 20% glycerol at −80 °C. During experimental work, they were grown in LA media and stored at 4 °C. For experimental purposes, overnight cultures were grown in the appropriate media at 37 °C with constant aeration in a shaker at 180 rpm. Strains were collected in the Institute of Microbiology and Immunology, University of Belgrade, Faculty of Medicine, where they have been previously identified and characterized [71].

### 4.3. Microdilution Assay

The microdilution assay was performed as it was described in [72]. The overnight bacterial culture was diluted to 10^6^ cell/mL, which was used in the subsequent steps of the experiment. The medium used in this experiment was the Mueller–Hinton broth (MHB), while the range of the tested CEO and EM was from 0.031 mg/mL to 4 mg/mL. Chloramphenicol was used as antibiotic control, followed by DMSO, which was used as solvent control. The experiment was performed in microtiter plates with 96 wells in 200 µL of the total volume. After the serial dilutions were prepared within the wells, the plates were incubated at 37 °C for 24 h. Resazurin solution, in a final concentration of 0.675 mg/mL, was added in each well followed by 3 h of reincubation until the color of the solution in the wells changed from blue to pink. The lowest tested concentration at which there were no color change in the resazurine was taken as the MIC value.

### 4.4. Antibiofilm Assay

#### 4.4.1. The Effect on Biofilm Formation

The experiment was performed in 96-well plates using TSB as a medium with added glucose in the final concentration of 0.5%, promoting biofilm formation. The tested concentrations of CEO and EM were in the range of 1/64 of the MIC values—MIC values, previously defined by microdilution assay. After the wells were filled with medium containing appropriate CEO and EM concentrations and bacterial inoculums (final concentration 10^6^ cell/mL), the plates were incubated for 24 h at 37 °C. The next day, the media were discarded carefully to prevent disturbing the formed biofilm. The wells were rinsed with water, after which, a solution of crystal violet (CV) stain was added. CV was incubated at room temperature for 15 min, after which, the plates were rinsed with tap water and left to dry. Ethanol was added in order to dissolve CV, and the optical density was measured on a 570 nm Multiskan Microplate Reader (Thermo Fisher Scientific, Waltham, MA, USA). The results of the inhibition of biofilm formation were calculated using the following formula:

$$\%\;\mathrm{biofilm}\;\mathrm{biomass}=\frac{ODsample}{ODcontrol}\times \;100\%$$

#### 4.4.2. The Effect on Pre-Formed Biofilm

The experiment was performed in 96-well plates using TSB as a medium and with 0.5% glucose added. The bacterial overnight culture was prepared to be in a final concentration of 10^6^ cell/mL in each well, and it was added in the wells in the final volume of 200 µL. The plates were incubated for 24 h at 37 °C. The next day, the wells were rinsed with sterile water and substituted with the same volume of fresh medium containing CEO and EM in the concentration range of ½ MIC—4 MIC. The plates were incubated for 24 h at 37 °C. After incubation, the media were discarded carefully in order to avoid disrupting the formed biofilms. The following procedure was the same as in Section 4.4.1.

### 4.5. Biofilm Matrix EPS Quantification

EPS quantification was performed as described by Tiwari et al. [42] and Rubini et al. [73] with slight modifications. A bacterial suspension (10^6^ cell/mL), prepared in TSB with 0.5% glucose, was transferred into a six-well plate (1 mL/well). After incubation, enabling biofilm formation for 24 h at 37 °C, the medium was replaced with the fresh one, containing CEO/EM in MIC concentrations, and additional incubation for 24 h at 37 °C was performed. Then, the medium was removed and 1.5M NaCl was added to scrape the biofilm. The obtained bacterial suspension was transferred into tubes, incubated for 10 min at 25 °C and centrifuged for 10 min, 5000× *g*. The supernatant (1/5 vol) was then transferred into tubes containing 1/5 vol 5% phenol solution, and 1 vol of conc. H_2_SO_4_ was added. After gentle mixing and an additional incubation for 10 min at 25 °C, the optical density was measured on a Multiskan Microplate Reader (Thermo Fisher Scientific, Waltham, MA, USA), using a wavelength of 492 nm. The same procedure, but without CEO/EM, was used to prepare the negative control, while the addition of the appropriate DMSO concentration was used as solvent control. Results were calculated and presented according to the following formula:

$$\%\;\mathrm{EPS}=\frac{ODSample}{ODControl}\times \;100\%$$

### 4.6. Biofilm Matrix eDNA Quantification

The isolation and quantification of eDNA was performed as described by Vuletić et al. [74]. Briefly, a biofilm was formed in 12-well plates (10^6^ cell/mL) for 24 h at 37 °C. After that, the medium was replaced with a fresh one containing CEO/EM in the MIC concentrations; the plates were further incubated at 37 °C for 24 h and the medium was replaced with 1 × PBS to scrape the biofilm. The obtained suspension was moved to tubes and Proteinase K (5 µg/mL) was added; further incubation for 2 h at 37 °C was performed. The samples were then centrifuged at 12,000 rpm, for 10 min, and the supernatants were transferred into new tubes. Then, 100% ethanol (2.5 vol) and 3M Na-acetate (1/10 vol) were added. After additional centrifugation under the same conditions and by discarding the supernatant, the residue was resuspended in TE buffer (15 µL). The concentration of isolated eDNA was measured using NanoDrop 2000C (Spectrophotometer, Thermo Scientific, Waltham, MA, USA).

### 4.7. Biofilm Matrix Protein Quantification

The biofilm used for protein quantification was formed in Petri dishes from 20 mL of bacterial inoculum (10^6^ cell/mL) prepared in TSB media containing 0.5% glucose. After 24 h at 37 °C, the incubation medium was replaced with a fresh one containing CEO/EM in MIC concentrations. Biofilm treatment was continued for another 24 h at 37 °C. After the medium was removed, the biofilms were rinsed and scraped with 1 × PBS. The suspension was transferred into tubes and centrifuged for 10 min, 5000× *g*, at 4 °C. After that, the supernatant was collected into new tubes and an appropriate volume of 50% TCA was added to adjust it to the final concentration of 20%. Then, the protein precipitation (24 h at 4 °C) tubes were centrifuged (14,000× *g*, 5 min, 4 °C). To purify the precipitated proteins, a procedure involving supernatant removement, the addition of ice-cold acetone (200 µL), and centrifugation under the same conditions was repeated twice. Finally, rehydration buffer (Na_2_HPO_4_·12H_2_O and NaH_2_PO_4_·2H_2_O solution, 0.1M, pH 6.8) was added to dissolve the precipitated proteins. For the protein quantification, the Bradford reagent was used. Optical density was measured at wavelengths of 595 nm on a Multiskan Microplate Reader (Thermo Fisher Scientific, Waltham, MA, USA) and the results were calculated according to the following formula:

$$\%\mathrm{Total}\;\mathrm{share}\;\mathrm{of}\;\mathrm{proteins}=100\;\times \;\frac{ODsample}{ODcontrol}$$

### 4.8. Motility Assay

The motility assay was performed as it was described by Raorane et al. [48]. Briefly, a TSB medium containing 0.25% agar and the selected concentrations of CEO/EM (1/2 MIC concentrations) was prepared. After medium constriction, 5 µL of fresh overnight culture was added to the center of medium. After incubation for 48 h on 37 °C, the motility areas were measured in software program ImageJ (ImageJ 1.38e/Java1.5.0_09).

### 4.9. RNA Isolation

The isolation of RNA molecules was performed as it was described by Đukanović et al. [75] with slight changes. The biofilm was prepared in 12-well plates, using 500 μL of cell suspension (10^6^ cell/mL). After incubation at 37 °C for 24 h, the medium was replaced with a fresh one containing the MIC of CEO/EM, and the plate was additionally incubated at 37 °C. After that, the RNA molecules were isolated. The medium was carefully pulled out from the well in order to avoid disturbing the formed biofilm. Trizol (500 µL) was added in each well, the plate was gently shaken, and the biofilm was scraped. The suspension was transferred into new tubes, vortexed for 20 s, and incubated for 5 min at room temperature. Chloroform (100 µL) was added, and the tubes were shaken for 15 s and additionally incubated for 2 min. The tubes were then centrifuged for 15 min (12,000× *g*, 4 °C). The upper faze was transferred into new tubes, and an addition of isopropanol (250 µL) was followed by vortexing, 10 min of incubation, and centrifugation for 10 min (12,000× *g*, 4 °C). The supernatant was discarded and the residue was resuspended in 75% ethanol. The tubes were vortexed and then centrifuged for 5 min at 4 °C, 7500× *g*. The supernatant was discarded and the last step was repeated. Then, the tubes were dried, 50 µL of PCR water was added, and an incubation for 5 min at 60 °C was performed. RNA purity and concentration were determined using NanoDrop 2000C Spectrophotometer (Thermo Fisher Scientific, Waltham, MA, USA). Translating RNA molecules into cDNA was performed with a commercially provided kit (RevertAid First Strand cDNA Synthesis Kit, Thermo Fisher Scientific, Waltham, MA, USA). The reaction was performed in a final volume of 12 µL and according to the manufacturer’s protocol.

### 4.10. RT-qPCR

Gene expression was determined using the RT-qPCR method (Step One Plus, Real Time PCR System, Applied Biosystems, Waltham, MA, USA). The final volume of each PCR mix per well was 12 µL (2 µL cDNA, specific 0.65 µL primers shown in Table 3, and PowerUp SYBR Green PCR Master Mix (Applied Biosystems by Thermo Fisher Scientific, Waltham, MA, USA)). We used 16S rRNA as the endogenous control, in relation to which the results were normalized. The conditions for the RT-qPCR reaction were as follows: 2 min at 50 °C, 10 min at 95 °C, 40 cycles at 95 °C for 15 s, and 1 min on 60 °C. The relative gene expressions were calculated according to the threshold (Ct).

### 4.11. Raman Spectroscopy

For Raman spectroscopy, biofilms were formed on quartz slides in Petri dishes containing 15 mL of bacterial suspension (10^6^ cell/mL) for 24 h at 37 °C. After that, the medium was replaced with the one containing CEO/EM (MIC concentrations). After incubation for 24 h at 37 °C, the slides were removed and left to dry out with constant air circulation for 2 h.

Raman spectroscopy was performed using the XploRA Raman spectrophotometer (Horiba Jobin Yvon) equipped with an Olympus BX 41 microscope (Olympus, Tokyo, Japan) where the samples of the *Acinetobacter* biofilm, without and with treatments, were measured. The wavelength of the laser Nd/YAG was 532 nm, grating 1200 lines/mm, resulting in spectra in the range of 350–1800 cm^−1^. Each sample was measured with an exposure time of 10 s and accumulated from 5 scans with a 100% filter. The spectral resolution was ~3 cm^−1^ and the calibration was checked using a 520.47 cm^−1^ line of silicon. Spectra acquisitions were carried out in LabSpec 6 software (Horiba Jobin Yvon, Palaiseau, France) and analyzed in Origin Pro 8.6. software (OriginLab, Northampton, MA, USA). Each sample was measured 10 times, and the mean values of the measured spectra are shown. The assignment of the main bands was based on the literature data mentioned in this section, as well as in Table 3 [62,63,64,65,66,67,68,69,70].

### 4.12. Statistical Analysis

Statistical analysis was performed using the software GraphPad Prism 6.01 (Software, Inc., San Diego, CA, USA). All the data were processed with the one-way ANOVA analysis using Dunnett’s test. The results were expressed as the mean value ± standard deviation. The threshold was adjusted to be * *p* < 0.05, ** *p* < 0.01, *** *p* < 0.001. The results of Raman spectroscopy were subjected to principal component analysis (PCA). PCA was performed on smoothed, baseline-corrected data normalized to the highest intensity band in the 350 cm^−1^ to 1800 cm^−1^ range. The spectra were preprocessed using Spectragryph software 1.2.14 (Menges, 2021 http://www.effemm2.de/spectragryph/, accessed on 27 October 2022) [80]. The spectra were base-corrected using Savitzky–Golay filters with 7 points and a second-order polynomial function was used for spectrum smoothing. PCA was performed using PAST software (http://palaeo-electronica.org/2001_1/past/issue1_01.htm, accessed on 15 May 2024) [81].

## 5. Conclusions

In conclusion, the significant antibacterial activity of CEO and the much stronger antibacterial effect of EM can be observed. The results obtained from the antibiofilm assays have revealed noteworthy antibiofilm efficacy against the tested *A. baumannii* clinical isolates, especially on preformed biofilm. Investigating CEO and EM activity on selected biofilm matrix components showed promising results, especially in the case of a notable decrease in protein share. Raman spectroscopy enhanced our knowledge regarding the biofilm matrix structure and provided the results concerning the potential types of the most dominant compounds. Furthermore, the PC analysis was able to attribute the differences between untreated and treated biofilms to certain constituents/chemical bonds that were affected by the treatments. Our understanding of the molecular pathways of CEO and EM antibiofilm activity needs to be improved by analyzing a wider spectrum of genes and their expression under the influence of CEO and EM in order to fully understand the possible targets affected by their antibiofilm properties. Taking everything into account, our future will concentrate on further analyzing the mechanisms of action of the promising CEO and EM antibiofilm agents.

## Figures and Tables

**Table 1:** eDNA concentrations (ng/μL) isolated from the *A. baumannii* clinical strain biofilm matrix.

	Control	DMSO	CEO	EM
GN 189	96.15 ± 59.75	141.55 ± 36.13	162.05 ± 6.57	225.85 ± 29.63
GN 242	391.5 ± 69.01	390.6 ± 5.09	318.95 ± 17.89	600.45 ± 98.92
GN 1105	38.5 ± 6.65	33.8 ± 3.73	53.3 ± 8.63	35.6 ± 2.55

**Table 3:** Primer sequences applied in this work.

Gene	Sequence	References
*aba*I	Forward—CCG CCT TCC TCT AGC AGT CA Reverse—AAA ACC CGC AGC ACG TAA TAA	[76]
*csu*A	Forward—TGG TAC AGC AGT AGC TTG GC Reverse—GAC GGT GGT GAA CGT ACA GA	[77]
*pil*A	Forward—TGT GGA TGA TGT GCC GGA AA Reverse—ATC CGG TAA GCA TCG GTG TG	[78]
16S rRNA	Forward—GCA ACG CGA AGA ACC TTA Reverse—AAC CCA ACA TCT CAC GAC AC	[79]

## Data Availability

All generated data during this study can be made available upon request.

## References

[1] Mohamed E.A., Raafat M.M., Samir Mohamed R., Ali A.E.E. (2023). *Acinetobacter baumannii* biofilm and its potential therapeutic targets. Future J. Pharm. Sci..

[2] Gedefie A., Demsis W., Ashagrie M., Kassa Y., Tesfaye M., Tilahun M., Bisetegn H., Sahle Z. (2021). *Acinetobacter baumannii* biofilm formation and its role in disease pathogenesis: A review. Infect. Drug Resist..

[3] Mea H.J., Yong P.V.C., Wong E.H. (2021). An overview of *Acinetobacter baumannii* pathogenesis: Motility, adherence and biofilm formation. Microbiol. Res..

[4] Tang J., Chen Y., Wang X., Ding Y., Sun X., Ni Z. (2020). Contribution of the AbaI/AbaR quorum sensing system to resistance and virulence of *Acinetobacter baumannii* clinical strains. Infect. Drug Resist..

[5] Flemming H.C., Wingender J. (2010). The biofilm matrix. Nat. Rev. Microbiol..

[6] El Kheloui Raja E.M.S., Asma L., Rachida M., Fatima H. (2022). *Acinetobacter baumannii* Extracellular Matrix as An Antibiofilm and Anti-Infection Target. World J. Pharm. Res..

[7] Reena A.A.A., Subramaniyan A., Kanungo R. (2017). Biofilm formation as a virulence factor of *Acinetobacter baumannii*: An emerging pathogen in critical care units. J. Curr. Res. Sci. Med..

[8] De Gregorio E., Del Franco M., Martinucci M., Roscetto E., Zarrilli R., Di Nocera P.P. (2015). Biofilm-associated proteins: News from Acinetobacter. BMC Genom..

[9] Campoccia D., Montanaro L., Arciola C.R. (2021). Extracellular DNA (eDNA). A major ubiquitous element of the bacterial biofilm architecture. Int. J. Mol. Sci..

[10] Pancu D.F., Scurtu A., Macasoi I.G., Marti D., Mioc M., Soica C., Coricovac D., Horhat D., Poenaru M., Dehelean C. (2021). Antibiotics: Conventional therapy and natural compounds with antibacterial activity—A pharmaco-toxicological screening. Antibiotics.

[11] Błaszczyk N., Rosiak A., Kałużna-Czaplińska J. (2021). The potential role of cinnamon in human health. Forests.

[12] Turek C., Stintzing F.C. (2013). Stability of essential oils: A review. Compr. Rev. Food Sci. Food Saf..

[13] Bakkali F., Averbeck S., Averbeck D., Idaomar M. (2008). Biological effects of essential oils—A review. Food Chem. Toxicol..

[14] Baptista-Silva S., Borges S., Ramos O.L., Pintado M., Sarmento B. (2020). The progress of essential oils as potential therapeutic agents: A review. J. Essent. Oil Res..

[15] Cimino C., Maurel O.M., Musumeci T., Bonaccorso A., Drago F., Souto E.M., Pignatello R., Carbone C. (2021). Essential oils: Pharmaceutical applications and encapsulation strategies into lipid-based delivery systems. Pharmaceutics.

[16] Ganić T., Vuletić S., Nikolić B., Stevanović M., Kuzmanović M., Kekić D., Cvetković S., Mitić-Ćulafić D. (2022). Cinnamon essential oil and its emulsion as efficient antibiofilm agents to combat *Acinetobacter baumannii*. Front. Microbiol..

[17] Silva N.B.S., Marques L.A., Röder D.D.B. (2021). Diagnosis of biofilm infections: Current methods used, challenges and perspectives for the future. J. Appl. Microbiol..

[18] Vázquez-López R., Solano-Gálvez S.G., Juárez Vignon-Whaley J.J., Abello Vaamonde J.A., Padró Alonzo L.A., Rivera Reséndiz A., Muleiro Alvarez M., Lopez E.N.V., Franyuti-Kelly G., Alvarez-Hernandez D.A. (2020). *Acinetobacter baumannii* resistance: A real challenge for clinicians. Antibiotics..

[19] Silva E., Teixeira J.A., Pereira M.O., Rocha C.M., Sousa A.M. (2023). Evolving biofilm inhibition and eradication in clinical settings through plant-based antibiofilm agents. Phytomedicine.

[20] Firmino D.F., Cavalcante T.T., Gomes G.A., Firmino N.C., Rosa L.D., de Carvalho M.G., Catunda Jr F.E. (2018). Antibacterial and antibiofilm activities of *Cinnamomum* sp. essential oil and cinnamaldehyde: Antimicrobial activities. Sci. World J..

[21] Asma S.T., Imre K., Morar A., Herman V., Acaroz U., Mukhtar H., Arslan-Acaroz D., Shah S.R.A., Gerlach R. (2022). An overview of biofilm formation–combating strategies and mechanisms of action of antibiofilm agents. Life.

[22] Intorasoot A., Chornchoem P., Sookkhee S., Intorasoot S. (2017). Bactericidal activity of herbal volatile oil extracts against multidrug-resistant *Acinetobacter baumannii*. J. Intercult. Ethnopharmacol..

[23] Mohamed S.H., Salem D., Azmy M., Fam N.S. (2018). Antibacterial and antibiofilm activity of cinnamaldehyde against carbapenem-resistant *Acinetobacter baumannii* in Egypt: In vitro study. J. Appl. Pharm. Sci..

[24] Cardoso-Ugarte G.A., López-Malo A., Sosa-Morales M.E., Preedy V.R. (2016). Cinnamon (*Cinnamomum zeylanicum*) essential oils. Essential Oils in Food Preservation, Flavor and Safety.

[25] Jayaprakasha G.K., Rao L.J.M. (2011). Chemistry, biogenesis, and biological activities of *Cinnamomum zeylanicum*. Crit. Rev. Food Sci..

[26] Mishra R., Panda A.K., De Mandal S., Shakeel M., Bisht S.S., Khan J. (2020). Natural anti-biofilm agents: Strategies to control biofilm-forming pathogens. Front. Microbiol..

[27] Millezi A.F., Costa K.A.D., Oliveira J.M., Lopes S.P., Pereira M.O., Piccoli R.H. (2019). Antibacterial and anti-biofilm activity of cinnamon essential oil and eugenol. Cienc. Rural.

[28] Liu F., Jin P., Sun Z., Du L., Wang D., Zhao T., Doyle M.P. (2021). Carvacrol oil inhibits biofilm formation and exopolysaccharide production of *Enterobacter cloacae*. Food Control.

[29] Kim Y.G., Lee J.H., Kim S.I., Baek K.H., Lee J. (2015). Cinnamon bark oil and its components inhibit biofilm formation and toxin production. Int. J. Food Microbiol..

[30] Prakash A., Baskaran R., Nithyanand P., Vadivel V. (2020). Effect of nanoemulsification on the antibacterial and anti-biofilm activities of selected spice essential oils and their major constituents against *Salmonella enterica* Typhimurium. J. Clust. Sci..

[31] Tapia-Rodriguez M.R., Cantu-Soto E.U., Vazquez-Armenta F.J., Bernal-Marcado A.T., Ayala-Zavala J.F. (2023). Inhibition of *Acinetobacter baumannii* biofilm formation by terpenes from Oregano (*Lippia graveolens*) essential oil. Antibiotics.

[32] Choudhary M., Shrivastava R., Vashistt J. (2022). Eugenol and geraniol impede Csu-pilus assembly and evades multidrug-resistant *Acinetobacter baumannii* biofilms: In-vitro and in-silico evidence. Biochem. Biophys. Res. Commun..

[33] Topa S.H., Subramoni S., Palombo E.A., Kingshott P., Rice S.A., Blackall L.L. (2018). Cinnamaldehyde disrupts biofilm formation and swarming motility of *Pseudomonas aeruginosa*. Microbiology.

[34] Artini M., Papa R., Barbato G., Scoarughi G.L., Cellini A., Morazzoni P., Bombardelli E., Selan L. (2012). Bacterial biofilm formation inhibitory activity revealed for plant derived natural compounds. Bioorg. Med. Chem..

[35] Selvaraj A., Valliammai A., Sivasankar C., Suba M., Sakthivel G., Pandian S.K. (2020). Antibiofilm and antivirulence efficacy of myrtenol enhances the antibiotic susceptibility of *Acinetobacter baumannii*. Sci. Rep..

[36] Albano M., Crulhas B.P., Alves F.C.B., Pereira A.F.M., Andrade B.F.M.T., Barbosa L.N., Furlanetto A., Lyra L.P.S., Rall V.L.M., Júnior A.F. (2019). Antibacterial and anti-biofilm activities of cinnamaldehyde against *S. epidermidis*. Microb. Pathog..

[37] Flemming H.C., Wingender J., Szewzyk U., Steinberg P., Rice S.A., Kjelleberg S. (2016). Biofilms: An emergent form of bacterial life. Nat. Rev. Microbiol..

[38] Subhaswaraj P., Barik S., Macha C., Chiranjeevi P.V., Siddhardha B. (2018). Anti quorum sensing and anti biofilm efficacy of cinnamaldehyde encapsulated chitosan nanoparticles against *Pseudomonas aeruginosa* PAO1. LWT.

[39] Fleming D., Chahin L., Rumbaugh K. (2017). Glycoside hydrolases degrade polymicrobial bacterial biofilms in wounds. Antimicrob. Agents Chemother..

[40] Fong J.N., Yildiz F.H., Ghannoum M., Parsek M., Whiteley M., Mukherjee P.K. (2015). Biofilm matrix proteins. Microbial Biofilms.

[41] Goh H.S., Beatson S.A., Totsika M., Moriel D.G., Phan M.D., Szubert J., Runnegar N., Sidjabat H.E., Paterson D.L., Nimmo G.R. (2013). Molecular analysis of the *Acinetobacter baumannii* biofilm-associated protein. Appl. Environ. Microbiol..

[42] Tiwari V., Tiwari D., Patel V., Tiwari M. (2017). Effect of secondary metabolite of *Actinidia deliciosa* on the biofilm and extra-cellular matrix components of *Acinetobacter baumannii*. Microb. Pathog..

[43] Banerji R., Mahamune A., Saroj S.D. (2022). Aqueous extracts of spices inhibit biofilm in *Listeria monocytogenes* by downregulating release of eDNA. LWT.

[44] Cui H., Zhang C., Li C., Lin L. (2020). Inhibition mechanism of cardamom essential oil on methicillin-resistant *Staphylococcus aureus* biofilm. LWT.

[45] Yamabe K., Arakawa Y., Shoji M., Miyamoto K., Tsuchiya T., Minoura K., Akeda Y., Tomono K., Onda M. (2022). Enhancement of *Acinetobacter baumannii* biofilm growth by cephem antibiotics via enrichment of protein and extracellular DNA in the biofilm matrices. J. Appl. Microbiol..

[46] Xi C., Wu J. (2010). dATP/ATP, a multifunctional nucleotide, stimulates bacterial cell lysis, extracellular DNA release and biofilm development. PLoS ONE.

[47] Nait Chabane Y., Mlouka M.B., Alexandre S., Nicol M., Marti S., Pestel-Caron M., Vila J., Jouenne T., Dé E. (2014). Virstatin inhibits biofilm formation and motility of *Acinetobacter baumannii*. BMC Microbiol..

[48] Raorane C.J., Lee J.H., Kim Y.G., Rajasekharan S.K., García-Contreras R., Lee J. (2019). Antibiofilm and antivirulence efficacies of flavonoids and curcumin against *Acinetobacter baumannii*. Front. Microbiol..

[49] McQueary C.N., Kirkup B.C., Si Y., Barlow M., Actis L.A., Craft D.W., Zurawski D.V. (2012). Extracellular stress and lipopolysaccharide modulate *Acinetobacter baumannii* surface-associated motility. J. Microbiol..

[50] He X., Lu F., Yuan F., Jiang D., Zhao P., Zhu J., Cheng H., Cao J., Lu G. (2015). Biofilm formation caused by clinical *Acinetobacter baumannii* isolates is associated with overexpression of the AdeFGH efflux pump. Antimicrob. Agents Chemother..

[51] Ramezanalizadeh F., Owlia P., Rasooli I. (2020). Type I pili, CsuA/B and FimA induce a protective immune response against *Acinetobacter baumannii*. Vaccine.

[52] Quinn B., Rodman N., Jara E., Fernandez J.S., Martinez J., Traglia G.M., Montana S., Cantera V., Place K., Bonomo R.A. (2018). Human serum albumin alters specific genes that can play a role in survival and persistence in *Acinetobacter baumannii*. Sci. Rep..

[53] Li H., Du X., Chen C., Qi J., Wang Y. (2022). Integrating transcriptomics and metabolomics analysis on kojic acid combating *Acinetobacter baumannii* biofilm and its potential roles. Microbiol. Res..

[54] Moon K.H., Weber B.S., Feldman M.F. (2017). Subinhibitory concentrations of trimethoprim and sulfamethoxazole prevent biofilm formation by *Acinetobacter baumannii* through inhibition of Csu pilus expression. Antimicrob. Agents Chemoter..

[55] Fernandez J.S., Tuttobene M.R., Montaña S., Subils T., Cantera V., Iriarte A., Tuchscherr L., Ramirez M.S. (2022). *Staphylococcus aureus* α-toxin effect on Acinetobacter baumannii behavior. Biology.

[56] Eijkelkamp B.A., Stroeher U.H., Hassan K.A., Papadimitrious M.S., Paulsen I.T., Brown M.H., Lo R. (2011). Adherence and motility characteristics of clinical *Acinetobacter baumannii* isolates. FEMS Microbiol. Lett..

[57] Corral J., Pérez-Varela M., Sánchez-Osuna M., Cortés P., Barbé J., Aranda J. (2021). Importance of twitching and surface-associated motility in the virulence of *Acinetobacter baumannii*. Virulence.

[58] Li M., Aye S.M., Ahmed M.U., Han M.L., Li C., Song J., Boyce J.D., Powell D.R., Azad M.A.K., Velkov T. (2020). Pan-transcriptomic analysis identified common differentially expressed genes of *Acinetobacter baumannii* in response to polymyxin treatments. Mol. Omics.

[59] Dhabaan G.N., AbuBakar S., Cerqueira G.M., Al-Haroni M., Pang S.P., Hassan H. (2016). Imipenem treatment induces expression of important genes and phenotypes in a resistant *Acinetobacter baumannii* isolate. Antimicrob. Agents Chemother..

[60] Martinez J., Fernandez J.S., Liu C., Hoard A., Mendoza A., Nakanouchi J., Rodman N., Courville R., Tuttobene M.R., Lopez C. (2019). Human pleural fluid triggers global changes in the transcriptional landscape of *Acinetobacter baumannii* as an adaptive response to stress. Sci. Rep..

[61] Ren D., Zuo R., González Barrios A.F., Bedzyk L.A., Eldridge G.R., Pasmore M.E., Wood T.K. (2005). Differential gene expression for investigation of *Escherichia coli* biofilm inhibition by plant extract ursolic acid. Appl. Environ. Microbiol..

[62] Kusić D., Kampe B., Ramoji A., Neugebauer U., Rösch P., Popp J. (2015). Raman spectroscopic differentiation of planktonic bacteria and biofilms. Anal. Bioanal. Chem..

[63] Shakeel M., Majeed M.I., Nawaz H., Rashid N., Ali A., Haque A., Akbar M.U., Tahir M., Munir S., Ali Z. (2022). Surface-enhanced Raman spectroscopy for the characterization of pellets of biofilm forming bacterial strains of *Staphylococcus epidermidis*. Photodiagn. Photodyn. Ther..

[64] Gieroba B., Krysa M., Wojtowicz K., Wiater A., Pleszczyńska M., Tomczyk M., Sroka-Bartnicka A. (2020). The FT-IR and Raman spectroscopies as tools for biofilm characterization created by cariogenic streptococci. Int. J. Mol. Sci..

[65] Chen Y.P., Zhang P., Guo J.S., Fang F., Gao X., Li C. (2013). Functional groups characteristics of EPS in biofilm growing on different carriers. Chemosphere.

[66] Kusić D., Kampe B., Rösch P., Popp J. (2014). Identification of water pathogens by Raman microspectroscopy. Water Res..

[67] Jung G.B., Nam S.W., Choi S., Lee G.J., Park H.K. (2014). Evaluation of antibiotic effects on *Pseudomonas aeruginosa* biofilm using Raman spectroscopy and multivariate analysis. Biomed. Opt. Express.

[68] Ivleva N.P., Wagner M., Horn H., Niessner R., Haisch C. (2009). Towards a nondestructive chemical characterization of biofilm matrix by Raman microscopy. Anal. Bioanal. Chem..

[69] Pezzotti G., Ofuji S., Imamura H., Adachi T., Yamamoto T., Kanamura N., Ohgitani E., Marin E., Zhu W., Mazda O. (2023). In Situ Raman Analysis of Biofilm Exopolysaccharides Formed in *Streptococcus mutans* and *Streptococcus sanguinis* Commensal Cultures. Int. J. Mol. Sci..

[70] Ramirez-Mora T., Dávila-Pérez C., Torres-Méndez F., Valle-Bourrouet G. (2019). Raman spectroscopic characterization of endodontic biofilm matrices. J. Spectrosc..

[71] Lukovic B., Gajic I., Dimkic I., Kekic D., Zornic S., Pozder T., Radisavljevic S., Opavski N., Kojic M., Ranin L. (2020). The first nationwide multicenter study of *Acinetobacter baumannii* recovered in Serbia: Emergence of OXA-72, OXA-23 and NDM-1-producing isolates. Antimicrob. Resist. Infect. Control.

[72] Tomić N., Stevanović M.M., Filipović N., Ganić T., Nikolić B., Gajić I., Ćulafić D.M. (2024). Resveratrol/Selenium Nanocomposite with Antioxidative and Antibacterial Properties. Nanomaterials.

[73] Rubini D., Banu S.F., Nisha P., Murugan R., Thamotharan S., Percino M.J., Subramani P., Nithyanand P. (2018). Essential oils from unexplored aromatic plants quench biofilm formation and virulence of Methicillin resistant *Staphylococcus aureus*. Microb. Pathog..

[74] Vuletić S., Ganić T., Lončarević B., Cvetković S., Nikolić B., Lješević M., Mitić-Ćulafić D. (2025). New insights into underlying mechanism involved in the *Frangula alnus* antivirulence potential directed toward *Staphylococcus aureus*. Bot. Serbica.

[75] Đukanović S., Ganić T., Lončarević B., Cvetković S., Nikolić B., Tenji D., Randjelović D., Mitić-Ćulafić D. (2022). Elucidating the antibiofilm activity of Frangula emodin against *Staphylococcus aureus* biofilms. J. Appl. Microbiol..

[76] Selasi G.N., Nicholas A., Jeon H., Na S.H., Kwon H.I., Kim Y.J., Heo S.T., Oh M.H., Lee J.C. (2016). Differences in biofilm mass, expression of biofilm-associated genes, and resistance to desiccation between epidemic and sporadic clones of carbapenem-resistant *Acinetobacter baumannii* sequence type 191. PLoS ONE.

[77] Lannan F.M., O’conor D.K., Broderick J.C., Tate J.F., Scoggin J.T., Moran N.A., Hussan C.M., Hegeman E.M., Ogrydziak C.E., Singh S.A. (2016). Evaluation of virulence gene expression patterns in *Acinetobacter baumannii* using quantitative real-time polymerase chain reaction array. Mil. Med..

[78] Priyadharsini J.V., Girija A.S., Paramasivam A. (2018). In silico analysis of virulence genes in an emerging dental pathogen *A. baumannii* and related species. Arch. Oral Biol..

[79] Wang Y., Li Y., Wang J., Wang X. (2018). FleQ regulates both the type VI secretion system and flagella in *Pseudomonas putida*. Biotechnol. Appl. Biochem..

[80] Menges F. Spectragryph Optical Spectroscopy Software, Version 1.2.14. http://www.effemm2.de/spectragryph/.

[81] Hammer O.Y.V.I.N.D., Harper D.A., Ryan P.D. (2001). Palaeontological statistics software package for education and data analysis. Palaeontol. Electron..

